# Asiatic Cholera—Precautionary Measures in Buffalo

**Published:** 1865-11

**Authors:** 


### Editorial Department.

### Asiatic Cholera---Precautionary Measures in Buffalo.

Since our last issue, a very well prepared report upon the dangers of cholera again visiting our city, and the proper means for mitigating its severity, was received by the Board of Health, from the Health Physician, Dr. Sandford Eastman; and we understand that vigorous measures are being adopted, for a general, thorough examination of the whole city, and a systematic removal of all causes and sources of disease which are found in operation within the city itself, as well as careful preparation for the appearance of any epidemic which may be feared from abroad. We hope this duty will be entrusted to vigorous hands, and that the work will be thoroughly done, that every nook and corner of the city will be washed and kept hereafter in cleanly condition. If this work is properly done, though it may not prevent cases of cholera appearing in Buffalo, yet we believe it will be the means of preventing such disease from becoming a general epidemic. There is one other, immense nuisance, vastly greater and more to be dreaded than the open cess-pools, drains and vaults which are so great sources of disease. The observation of many authors, goes to show that beer and whisky drinking is *the most fruitful source* of cholera—that during an epidemic of this disease, this habit determines perhaps more than any other, who shall be the victims; those poor, unfortunate subjects of intemperance become the easy and natural prey of epidemic disease.

This fact should be placed before the public, that all may understand how that they must not only, not eat improper food, but also must not drink to intoxication. The history of the last epidemic of cholera shows in some cities, that many of those who drank freely Saturday and Sunday evenings (a common habit) died early Monday morning.

It might also be well to assure the public that though no hygienic conditions could insure absolute immunity from such disease during its epidemic prevalence, still very little is to be feared by those who avoid the known and common exciting causes. Fear is one of these causes, and stands in potency next to unclean ness and intemperance. Cholera has not yet made its appearance in Buffalo, and it is by no means certain that it will, if it should, it may only appear in mild type, and produce but rarely fatal results—never unless aggravated by the influences indicated.

It is well to stimulate to active and energetic hygienic and preventive measures, but it is desirable that when this has been accomplished the excited fears in the public mind be also quieted, since alarm and fear are no less active agents in inducing disease than open sewers, overflowing vaults and lager beer; this dread in the public mind is not to be treated lightly; it is an important element and worthy of consideration. It has been already anticipated by charlatans and imposters, and cholera specifics and preventives are already announced. Vast sums of money will be early invested in this enterprise, and the "advertisements" would make the poor dupes who read them, believe that they are so potent as to even prevent the disease from crossing the Atlantic, if they are only kept in every dwelling and pocket ready for immediate use.

The history of the last epidemic of cholera goes to prove, that local and hygienic influences, are almost controlling—that the disease attacks for the most part only those who in some way provoke it, and that the other portion of the community suffer, if at all, mainly, from being "found in bad company."

